# The Role of Skin Substitutes in the Therapeutical Management of Burns Affecting Functional Areas

**DOI:** 10.3390/medicina61060947

**Published:** 2025-05-22

**Authors:** Matei Iordache, Luca Avram, Ioan Lascar, Adrian Frunza

**Affiliations:** 1 Department of Plastic Surgery and Reconstructive Microsurgery, “Carol Davila” University of Medicine and Pharmacy Bucharest, 010825 Bucharest, Romania; matei.iordache@umfcd.ro (M.I.);; 2 Burn Centre, Emergency Clinical Hospital of Bucharest, 014461 Bucharest, Romania

**Keywords:** burns, skin substitutes, dermal matrices, cultured skin substitutes

## Abstract

Considered one of the most severe types of trauma with a high impact upon patient survival, burns are the leading cause of disability-adjusted life-years (DALYs), and are responsible for high morbidity, prolonged hospitalization, disfigurement and social stigma. Of particular interest are injuries that affect the functional areas: face, neck, hand and fingers, joints, feet and soles and perineum. Burns to these regions highly influence the day-to-day activities of patients due to the formation of vicious scars and contractures, which may affect both quality of life and functional capacity. One of the primary challenges in the management of burn patients is the effective coverage of tissue defects resulting from such injuries. Cases that have a large area of burned surface also have a limited amount of total available skin. As such, the importance of skin substitutes increases, particularly in the treatment of these areas. Skin substitutes are widely utilized in plastic surgery due to their ability to promote wound healing by providing an extracellular matrix. Consequently, ongoing research has focused on developing skin substitutes that can serve as alternatives to autografts, addressing the challenges associated with large-scale tissue loss. This article aims to present and compare the most used skin substitutes, highlighting their respective advantages and limitations. This topic continues to be a subject of significant debate, as an ideal substitute has yet to be created. The cost–efficiency ratio is a practical consideration that must be tailored to each specific medical system. The available data in the literature usually present general guidelines, not rules, and as such, they need to be adapted to each patient’s necessities.

## 1. Introduction

As one of the most severe types of trauma with a high impact upon patient survival, burns are the leading cause of disability-adjusted life-years (DALYs), and are responsible for high morbidity, prolonged hospitalization, disfigurement and social stigma [1]. Worldwide, in a study conducted in 2023, it was found that around 180,000 deaths were caused by burns every year, with low- and middle-income countries being the most affected. Burns require immediate specialized care to minimize the damage and to prevent complications, especially as the skin has been proven to be a powerful neuro–immuno–endocrine organ that communicates with systemic centers in a bidirectional fashion. As such, the severity of the burns depends on their depth, extent and location [2,3].

Of particular interest are injuries that affect functional areas: the face, the neck, the hand and fingers, the joints, the feet and soles and the perineum. Burns to these regions highly influence the day-to-day activities of patients due to the formation of vicious scars and contractures, which may affect both quality of life and functional capacity.

Burn scar contractures develop, in part, due to the replacement of the skin’s naturally flexible tissue with scar tissue that lacks the necessary elasticity. Areas of the skin that are linked to joint range of motion (ROM) are especially vulnerable to developing contractures, which restrict joint movement. This limitation in ROM can lead to deformity, functional impairment, and long-term disability [4].

One of the primary challenges in the management of burn patients is the effective coverage of tissue defects resulting from such injuries. In referral centers, patients frequently have severe burns involving extensive surface areas and varying depths. Most burns are classified as deep partial-thickness (Grade IIB) and full-thickness (Grade III) burns. Given the substantial tissue loss in these cases, there is a pressing need to identify and implement the most effective methods for wound coverage, particularly when the patient’s own donor site resources are limited. Cases with a large area of burned surface also have a limited amount of total available skin. As such, treatment of functional areas should be prioritized with the best quality skin, which frequently means using full thickness skin grafts [5,6].

Skin substitutes are widely utilized in plastic surgery due to their ability to promote wound healing by providing an extracellular matrix. These substitutes may be derived from human (allografts), animal (xenografts) or biosynthetic sources. They are particularly valuable in cases involving extensive tissue loss, where autografts are insufficient. By serving as both a protective barrier against infection and a supportive environment for tissue regeneration, skin substitutes facilitate wound healing, reduce scarring and are especially beneficial in the management of burns and chronic wounds [7,8].

While autologous skin grafting remains the only definitive method for permanent wound coverage, it is often insufficient in cases of extensive burns due to limited donor site availability. As such, use of skin substitutes has increased in importance, especially in the case of these areas where it is even more important to prevent vicious scars from forming. The downside of these products is their high cost and the fact that in some cases they may not be easily accessible. For this reason, a good knowledge and understanding of their characteristics and function are essential, as they are used mostly in functional areas or in cases where there is higher need, with the aim of improving both functional and aesthetic outcomes [8].

Consequently, ongoing research has focused on developing skin substitutes that can serve as alternatives to autografts and address the challenges associated with large-scale tissue loss [9]. This article aims to present and compare the most used skin substitutes, highlighting their respective advantages and limitations.

The standard treatment for extensive deep dermal and full-thickness burns currently involves the excision of necrotic tissue followed by wound closure using a split-thickness skin graft (STSG) [10]. This approach primarily depends on autografts, where healthy skin is harvested from an unaffected donor site on the patient and transplanted onto the debrided wound bed.

In the management of acute burn wounds, split-thickness skin grafts remain the gold standard. Nevertheless, their utility is limited by the total body surface area (TBSA) that can be effectively covered. When burns involve more than 25% of the TBSA, the use of skin substitutes becomes essential. A significant advancement in this field was the development of the first artificial dermal skin substitute for extensive burn injuries, pioneered by Yannas and Burke [11].

This review is intended to be a comprehensive up-to-date presentation of dermal substitutes with their corresponding clinical indications. Since their discovery in the second half of the past century, they have increasingly become an essential tool in burn care in the acute phase of treatment, while also improving functional and cosmetic results with long-term quality-of-life enhancement. In the chronic setting, these substitutes are useful in reconstruction as well as in the improvement of burn scars and defects [12,13].

The challenges associated with poor scar quality and donor site morbidity following autologous split-thickness skin grafts (STSGs), coupled with the limited availability and associated morbidity of full-thickness grafts (FTGs), have underscored the importance of dermal regeneration in achieving optimal long-term outcomes. These issues have driven the continued advancement of skin replacement therapies [14].

## 2. Overview and Classification of Dermal Substitutes

In the past decade, dermal substitutes have gained prominence as an adjunctive treatment for deep dermal and full-thickness burns, offering additional options for wound management and reconstruction. They are able to reliably replicate the qualities of native skin while remaining economically feasible even in the poorest of settings [9,15,16,17].

The primary role of these materials is to facilitate the healing of deep skin wounds, although they may also be beneficial in certain superficial injuries. As three-dimensional structures, these tissue scaffolds must possess several essential properties. They should provide protection against fluid and protein loss; be non-antigenic, flexible and durable; prevent microbial and toxin infiltration from the external environment; and minimize pain while allowing sufficient time for either the donor site or the wound bed to heal. Additionally, some of these materials have the capability to absorb exudate, further contributing to an optimal wound healing environment. Unfortunately, an ideal substitute meeting all the ideal qualities has yet to be described [10,18,19,20].

Following the application of these tissue scaffolds, their integration into the wound bed occurs through natural healing processes within the body, primarily driven by inflammation, neovascularization, and cellular infiltration. Traditionally, these scaffolds have been acellular; however, advancements in biomedical technology have enabled the incorporation of living cells, such as keratinocytes and fibroblasts, into their structure. This has created a variety of products of different compositions and sources, as shown in Table 1. This innovation aims to enhance the regenerative capacity of the scaffolds and improve wound healing outcomes and tissue regeneration [21].

Based on their application method, dermal substitutes can be classified as either single-stage or two-stage. In the single-stage approach, the dermal substitute is applied directly to the wound bed and immediately covered with a split-thickness skin graft (STSG) in a single procedure. In contrast, the two-stage method involves the initial application of the dermal substitute, which is then temporarily covered with a sealing agent. This temporary coverage allows for integration and neovascularization of the substitute before a definitive closure with an STSG is performed at a later stage [12].

Another classification of skin substitutes, arranged in increasing order of complexity, includes three main types: acellular skin substitutes, cellular allogeneic skin substitutes, and cellular autologous skin substitutes [8]. The primary application of the major skin substitutes currently available on the market are summarized in Table 2.

### 2.1. Acellular Skin Substitutes

#### 2.1.1. Alloderm^®^

The potential application of allograft donor skin as a permanent replacement for full-thickness burns is constrained by its immunogenic properties. While allograft skin grafts are typically accepted by a full-thickness wound, they are ultimately rejected [38].

This immune response is primarily directed against the epidermal cells, as well as the endothelial and fibroblast cells within the dermis. In contrast, the non-cellular components of the dermis, primarily consisting of extracellular matrix proteins and collagen, have been shown to be relatively non-immunogenic. The challenge of isolating the immunogenic cells from the non-immunogenic dermal matrix has historically limited the use of allograft skin to temporary coverage of full-thickness burns [22].

Developed in 1992, cadaveric dermis is processed for burn injuries by removing the epidermal layer using salt solutions, followed by treatment with a non-denaturing detergent to create an acellular matrix [39,40]. This process eliminates cellular material, reducing the risk of immune rejection. The resulting biocompatible material preserves the basement membrane, collagen and elastin, offering a reliable scaffold for burn treatment. Key advantages include ease of application, time efficiency, aesthetic outcomes and the ability to treat multiple defects in one stage without requiring donor sites [39,41].

#### 2.1.2. Integra^®^

Integra^®^ artificial skin is the most commonly utilized synthetic skin substitute and is reported to yield better outcomes in terms of both appearance and elasticity when compared to split-thickness skin grafting (STSG) [42]. It is a bi-layered regeneration template that was initially developed for the acute phases in the burn patient’s evolution [43].

The dermal layer of Integra^®^ consists of bovine-derived collagen combined with shark chondroitin-6-sulphate, a glycosaminoglycan (GAG) forming a scaffold. The epidermal layer is a thin silicone sheet. The host’s response involves fibroblast migration, which initiates collagen production within this scaffold, followed by the infiltration of endothelial cells that work to establish a vascular network [44].

After a period of 2 to 4 weeks, the epidermal layer of Integra can be removed and replaced with an autologous thin split-thickness skin graft (STSG). However, some drawbacks of Integra^®^ include the risk of infection beneath the membrane, frequent dressing changes, the need for experienced placement, at least two surgical procedures, and the relatively high cost of the product [45].

It has been observed that when objectively measured, Integra^®^-treated sites show a stronger correlation to normal skin compared to autologous skin grafts, particularly in terms of elastic function and gross elasticity. The correlation coefficients between Integra^®^ and normal skin are notably higher, highlighting its superior performance in these aspects [42].

#### 2.1.3. Biobrane^®^

Biobrane^®^ is a flexible biosynthetic bilaminar skin substitute. The dermal layer is made of a nylon mesh, covered by a silicone membrane acting as the epidermis. It also contains porcine-derived collagen, which improves its adherence to the wound bed [46,47].

While typically used as a temporary solution until definitive skin grafting, Biobrane^®^ offers advantages such as reduced healing time, pain, and hospital stay [48].

However, similar to Integra^®^, issues with drainage may lead to infection, and, in some cases, toxic shock syndrome. However, some studies contradict this, suggesting that its permeability may help prevent this [48,49].

#### 2.1.4. Matriderm

Matriderm^®^ is a single-layer dermal matrix composed of bovine collagen and elastin hydrolysate designed to support dermal regeneration in full-thickness wounds. One of its significant advantages is its capacity to enable immediate split-thickness skin grafting, which facilitates enhanced neovascularization. It is primarily applied in the treatment of full-thickness skin defects, including those resulting from burn excision, and is particularly favored in aesthetically sensitive areas due to its regenerative properties.

In a comparative study by Dickson et al., a key difference observed between Matriderm^®^ and Integra^®^ was the rate of matrix resorption. Matriderm^®^ demonstrated early resorption beginning in the fourth postoperative week, with near-complete degradation within two months. In contrast, remnants of the Integra^®^ matrix remained histologically detectable up to two years post-surgery. This variance is attributed to the structural characteristics of the matrices: Integra^®^ is cross-linked, which maintains its integrity over time and supports gradual cellular infiltration. Conversely, Matriderm^®^ is non-cross-linked, promoting rapid cellular integration and wound bed assimilation [50].

In the context of hand burns, Matriderm^®^ has shown promising clinical outcomes. It has been demonstrated to support restoration of hand function, natural pliability, and improved aesthetic appearance when compared to split-thickness skin grafting alone. According to Dantzer et al., Matriderm^®^ is easy to handle and does not necessitate additional procedures during the healing phase. While the time to wound closure is comparable to grafting alone, Matriderm^®^ offers the added benefits of enhanced scar quality and long-term functional and cosmetic outcomes [51].

### 2.2. Cellular Allogeneic Skin Substitutes

The second major category of skin substitutes consists of cellular allogeneic products. Unlike the previously described materials, this class typically includes bilaminar structures populated with viable fibroblasts, often derived from neonatal foreskin. The presence of these cellular components enhances their biological activity, contributing to wound healing and tissue regeneration [8].

#### 2.2.1. Apligraf^®^

Apligraf^®^ is a bioengineered skin substitute composed of both dermal and epidermal components of living origin. Its production involves a two-step process. The first step entails the formation of a loose extracellular matrix by combining type I collagen of bovine origin with neonatal fibroblasts. This mixture is subjected to thermal processing, facilitating scaffold formation. Over the course of two weeks, these components develop into a dense fibrous network. In the second step, keratinocytes derived from neonatal foreskin are seeded onto the preformed scaffold, where they proliferate and differentiate over four days [8].

During the final two days, calcium concentration is increased, promoting the maturation of the keratinocyte layer and the formation of a stratum corneum. The entire maturation process typically requires 7 to 10 days, after which the product is ready for clinical application [52,53].

Although Apligraf^®^ has been shown to enhance wound healing, its precise mechanism of action remains incompletely understood. However, it is well established that the product induces the release of a significant number of growth factors and cytokines, which stimulate cellular proliferation and differentiation, even in chronic, non-healing wounds [54].

Multiple studies have reported that skin substitutes containing living cells facilitate a higher-quality healing process, characterized by a significant reduction in fibrosis. This effect is believed to be primarily attributed to the neonatal component of the product, which may play a crucial role in modulating the wound healing response [37].

In addition to its numerous advantages, several studies have demonstrated the effectiveness of Apligraf^®^ in enhancing the integration of meshed autografts when applied over them. The use of this product has been associated with improved functional and aesthetic outcomes compared to standard treatments. Furthermore, the study highlights its superior vascularity, with results nearly three times better than those observed in the control group, as well as enhanced pigmentation and pliability [55].

#### 2.2.2. TransCyte^®^

TransCyte^®^ is a product made of two layers—a nylon mesh covered by a silicone stratum mixed with neonatal foreskin, which promotes the formation of a collagen matrix and recruitment of growth factors over a period of 3–6 weeks [49,56].

The wound is either treated surgically at a later stage, or the natural detachment of the product from the wound bed is allowed to occur, which serves as an indication of underlying wound healing [8,57].

A study conducted by Kumar et al. in 2004 [58] compared the efficacy of three different products—TransCyte^®^, Biobrane^®^, and Silvazine^®^—in the treatment of partial-thickness burns. The study involved 58 wounds, with participants divided into three approximately equal groups, each receiving one of the three treatments. The results revealed that TransCyte^®^ demonstrated the shortest mean re-epithelialization time at 7.5 days, compared to 9.5 days for Biobrane^®^ and an even longer period for Silvazine^®^. Additionally, the number of wounds requiring autografting was significantly lower in the TransCyte^®^ group [58].

A study by Noordennbos et al. demonstrated that this product was easy to apply and manage for partial-thickness wounds. After 24–48 h, the wound was left exposed to air. Its transparency facilitated continuous monitoring, offering the advantage of easy detection of any fluid accumulation. TransCyte^®^ adhered well to the wound and could be removed without difficulty as the wound epithelialized, causing minimal discomfort to the patient [28].

#### 2.2.3. Dermagraft

Dermagraft is a product similar to TransCyte^®^, formed of a dermal layer—in this case an absorbable structure made of polyglycolic acid, but without the outer silicone layer [8]. It is, however, seeded with fibroblasts derived from neonatal foreskin. This product can be utilized independently or as a scaffold for split-thickness autografts, as well as for the temporary or permanent coverage of excised burn wounds [59,60]. Following placement, the mesh is gradually absorbed over a period of three to four weeks, during which the fibroblasts produce a collagen matrix, extracellular matrix proteins, cytokines and growth factors [61].

In addition, the product offers several advantages, including resistance to tearing, the absence of reported adverse reactions and no evidence of rejection [62].

### 2.3. Cellular Autologus Skin Replacements

Cellular autologous skin replacements represent advanced therapeutic strategies for the management of extensive skin injuries, including burns and chronic wounds. These substitutes are derived from the patient’s own cells, facilitating the creation of skin grafts that reduce the likelihood of immune rejection and enhance seamless integration with the recipient tissue [63].

While the previously described products offer significant benefits, they have a major limitation: most are typically used as temporary solutions for covering defects. As a result, they often require subsequent skin grafting or, in cases where the remaining wound is sufficiently small, healing by secondary intention [8]. This led to the development of autologous keratinocytes, which were first cultured by Rheinwald and Green [53].

Cellular autologous skin replacements come in two classifications: cultured epidermal autografts (CEA) and cultured skin substitutes (CSS).

#### 2.3.1. Cultured Epdiermal Autograft (CEA)

Cultured epithelial autografts (CEAs) have been utilized in burn therapy since 1981. A small biopsy of healthy skin is used to culture keratinocytes, which are then expanded into sheets over several weeks. In the mid-1980s, Cuono demonstrated successful graft take in full-thickness wound beds by combining cultured epithelial autografts with split-thickness allografts [64]. However, CEAs require a delivery vehicle or supportive dressing due to their high cost, difficulty in handling and unpredictable uptake. Additionally, because the dermal–epidermal interface of CEAs is not fully developed, friction-induced blister formation is a common complication. This approach has also been associated with several potential side effects, such as scarring, contracture and hyperkeratosis [65].

The process is incomplete because, after the biopsy is taken from the patient, the deeper layers, including the dermis and subcutaneous tissue, are removed. As a result, only the epidermal keratinocytes remain, which are then processed and cultured on irradiated mouse fibroblast layers. After several weeks of culture, these keratinocytes can be manipulated and used. However, the resulting graft is very fragile, as it lacks a basement membrane. Consequently, friction or mechanical stress can lead to the formation of blisters [8].

Another important consideration is the potential action of collagenase enzymes from the wound bed, which may compromise the successful integration of the substitute. To address this, some studies have proposed the use of allogenic skin harvested from cadavers, leaving it in place for at least four days before replacing it with the autologous substitute. This approach has shown success in enhancing graft take and promoting better outcomes [66].

#### 2.3.2. Cultured Skin Substitutes

Cellular skin substitutes (CSS) are a type of autologous graft that includes both the epidermal and dermal layers. They provide a permanent covering, possess a well-formed dermal–epidermal junction and are relatively simple to apply. CSS has demonstrated clinical outcomes comparable to autograft skin tissue, reducing the need for donor skin autografts in wound treatments. Additionally, CSS contributes to lower morbidity and mortality rates in the treatment of burns, chronic wounds and dermal reconstruction. However, the use of CSS is associated with higher costs and longer production times [65].

To obtain such a substitute, split-thickness skin must be harvested as soon as possible after the injury to allow sufficient time for laboratory processing. From this specimen, keratinocytes are isolated from the epidermal layer, while fibroblasts are extracted from the dermal layer. Both cell types must be cultured on selective media. Once enough fibroblasts are obtained, they are collected and placed onto a collagen-GAG (glycosaminoglycan) mesh, which is then incubated for more than 18 h. Similarly, the keratinocytes are isolated and inoculated, allowing them to incubate for 2 days. On the third day, the substitute is elevated to the interface between air and liquid, stimulating the formation of the epidermal barrier. Typically, after 10 to 14 days, the substitute is ready to be used for covering burn wounds [67,68,69].

They have several advantages: they are a permanent coverage solution; being autologous, the risk of infection transmission is minimal; they can be easily manipulated; and the basement membrane is stable so no blister formation is noticed [8]. The primary disadvantage, however, lies in the cost and the time required to produce the substitute.

One example of a promising product in this category is *PermaDerm*, which exhibits all the advantages previously discussed [65].

## 3. Proposed Guidelines for the Clinical Use of Skin Substitutes

Although it is obvious that the present available data is insufficient, as most of the works are reviews, these data were unable to prove the significant impact that dermal substitutes have on scarring when compared to the conventional split thickness skin grafts. More research is required. Although the subject of dermal substitutes is a relatively new and the subject of on-going research, several guidelines or indications can already be derived, as depicted in Table 3. Of course, these are general guidelines, not rules and as such, they should be adapted to each patient’s necessities in terms of the resources of the burn center.

## 4. Socioeconomical Considerations for the Use of Dermal Substitutes

This subject is an important one and requires special attention, as 85% of all major burns and 90% of fire-related deaths have been proven to occur in low- and middle-income countries (LMICs). This aspect is even more important as these countries are largely lacking in skin banking or substitute facilities [77]. Aside from this, unfortunately the available data is even scarcer. Records are easily available in high-income countries, while lower-income countries do not collect or publish their results as thoroughly. As such, the estimated incidence in Sub-Saharan Africa or in South Asia is around 10,000–14,000 deaths. Since the incidence is so high, and the problem is so severe, the use of dermal substitutes may become even more pressing in such instances. LMICs from the Eastern Mediterranean, Europe and the Americas report that burns caused by fire are the primary cause of disability-adjusted life years leading to an equivalent of loss of 1 year of good health. They report more than 30% DALYs in men aged 15–44 due to disability or death following the injury [78,79].

A study performed by Gupta reviewed 458 hospitals and showed that most of the hospitals in LMICs were able to correctly perform initial burn management as well as resuscitation but lack the capacity to further conduct treatment correctly in terms of skin grafting and management of the complications that might result from burns [80]. Another study of 1337 health facilities conducted in 32 LMICs noted that only 379 units, or 36.6%, were able to perform skin grafting. Only half of these had access to blood banks, i.e., only 18% of the initial facilities with the capacity for excision and early grafting. In this setting it is even less surprising that the number of hospitals with access to skin banks or the use of skin substitutes is extremely low [81,82].

Although the literature is scarce on the economical aspect of the use of dermal substitutes, a comprehensive study was performed by Hop et al. which sought to demonstrate the efficiency of using these materials and their enhancement of graft take, while also evaluating the financial aspect to complete the assessment of these products. As such, the patients in their study had deep dermal and full-thickness burns, which were eventually grafted. They were separated into four groups depending on the adjuvant methods employed in the process: dermal substitutes and negative pressure therapy, only dermal substitutes, only negative pressure and simple skin grafting. One clinical way to evaluate the effectiveness was by the quality of scar elasticity at 12 months. The best result was undoubtedly in the first group. These researchers performed a thorough evaluation of all the factors that influence cost, but when it comes to the materials used, they reported a cost of 864€ per patient treated by dermal substitutes and negative pressure (with a cost of 333€ per sheet of Matriderm—used for ~1% TBSA), 771€ for those treated just with dermal substitutes, 297€ in the negative pressure group and 425€ for the grafted group. They do report that out of the total costs, when taken individually, these products increase these values the most. Even so, they contribute to just 12% of the specialized care of the burns, and up to 7% of the total estimated costs [83,84]. These costs are even higher when it comes to the use of cellular substitutes, such as those containing human fibroblasts, which lead to costs of 18,430$ per patient. However, they are proven to lead to improved wound healing and quality-adjusted life years [85]. These costs may explain the lack of data from LMICs when it comes to the use of such products in the treatment of severe burns.

## 5. Limitations and Future Directions

The main limitation of this paper is the paucity of data from countries that have the highest exposure to burn traumas (LMICs) but also have the least available resources. Therefore, the results come mostly from high-income countries. Another issue is the potential bias in reporting in certain studies, as some studies may be influenced by conflicts of interest. Some articles reported on dermal substitutes, while others infrequently detailed the possible negative results.

The use of dermal substitutes in burns could benefit more from new studies comparing several options in the same class and reporting the cost-effectiveness of each product. This is an especially important aspect in low-income countries.

## 6. Conclusions

Severe burns remain a formidable clinical and socioeconomic challenge, in particular those affecting functional and aesthetically sensitive regions, which often exhaust a patient’s limited amount of skin. In such cases, autografting becomes insufficient, and so the need arises to use modern dermal substitutes. These offer faster defect coverage, scaffold-guided regeneration and superior pliability and scar quality versus skin grafts alone. Evidence favors an anatomy-specific protocol with thin elastic substitutes for the face and neck, sturdy collagen-GAG matrices for joints, hands and weight-bearing soles and fat-enriched or silicone-containing products for flexibility and cushioning. Access is limited in low- and middle-income countries due to the expense and sparse skin-bank infrastructure, despite the fact that these regions bear the greatest burn burden. Future progress depends on lowering manufacturing costs, simplifying application and conducting organized multicenter trials, particularly in resource-constrained environments with transparent reporting of functional outcomes, complications and economic impact. Until then, optimal burn care remains a patient-specific algorithm that matches substitute properties to wound biology, prioritizes functional areas and aligns with available resources to restore form and function while reducing global disparities.

## Figures and Tables

**Table 1:** Overview of Commonly Described Products with Composition and Source Information [20,22,23,24,25,26,27,28,29,30].

Category	Product Name	Composition	Source
I. Acellular Skin Substitutes	AlloDerm^®^ LifeCell Corporation, Branchburg, NJ, USA	Human dermal matrix	Human
	Integra^®^ Integra LifeSciences Corporation, Plainsboro, NJ, USA	Bovine collagen and glycosaminoglycan	Bovine
	Biobrane^®^ Smith & Nephew, Fort Worth, TX, USA	Nylon mesh and porcine collagen	Porcine
	Matriderm^®^ MedSkin Solutions Dr. Suwelack AG, Billerbeck, Germany	Bovine collagen and elastin hydrolysate	Bovine
	Human Amnion Various tissue banks; commonly sourced from local hospital tissue banks	Amniotic membrane	Human placenta
II. Cellular Allogenic Skin Substitutes	Apligraf^®^ Organogenesis Inc., Canton, MA, USA	Bovine collagen and human fibroblasts	Bovine and Human
	Transcyte^®^ Organogenesis Inc., Canton, MA, USA.	Human keratinocytes on a bioengineered scaffold	Human
	Dermagraft Organogenesis Inc., Canton, MA, USA.	Similar to Trascyte but without a silicon layer	Human
	StrataGraft^®^ Stratatech (a Mallinckrodt company), Madison, WI, USA	Cultured allogeneic keratinocytes and dermal fibroblasts	Allogeneic
III. Cellular Autologous Skin Replacements	Epicel^®^ Vericel Corporation, Cambridge, MA, USA	Cultured autologous epidermal cells	Autologous

**Table 2:** Overview of Product Types and Their Main Indications [31,32,33,34,35,36,37].

Product	Primary Use
AlloDerm^®^ LifeCell Corporation, Branchburg, NJ, USA	- Chronic wounds and burns - Hernia repair - Dental/periodontal surgery - Ophthalmic surgery
Integra^®^ Integra LifeSciences Corporation, Plainsboro, NJ, USA	- Full-thickness burns, chronic ulcers, traumatic skin loss - Scaffold for dermal regeneration - Neurosurgical use: dural repair and cranial reconstruction
Biobrane^®^ Smith & Nephew, Fort Worth, TX, USA	- Partial-thickness burns - Donor site coverage - Pain reduction and wound monitoring
Matriderm^®^ MedSkin Solutions Dr. Suwelack AG, Billerbeck, Germany	- Burn reconstruction - Post-traumatic wounds - Scalp reconstruction with exposed bone/tendons
Human Amnion Various tissue banks; commonly sourced from local hospital tissue banks	- Partial-thickness burns - Superficial wounds
Apligraf^®^ Organogenesis Inc., Canton, MA, USA	- Chronic wounds: Venous leg ulcer and Diabetic foot ulcer - Stimulates tissue regeneration - Not indicated for acute burns
TransCyte^®^ Organogenesis Inc., Canton, MA, USA	- Partial-thickness burns-Donor site coverage-Chronic wounds (select cases)
Dermagraft Organogenesis Inc., Canton, MA, USA.	- Extensive burns - Scaffold for split-thickness autografts
StrataGraft^®^ Stratatech (a Mallinckrodt company), Madison, WI, USA	- Deep partial-thickness thermal burns
Epicel^®^ Vericel Corporation, Cambridge, MA, USA	- Extensive deep dermal or full-thickness burns - Good when limited donor site availability - Autologous cultured epidermal grafts

**Table 3:** Distribution of Frequently Recommended Products by Functional Area [9,51,70,71,72,73,74,75,76].

Anatomical Region	Importance	Challenges	Treatment Approaches
Face	Highly vascularized means heals well; a good result is crucial for identity, communication, and expression. Function is just as important as aesthetic.	Scar formation can impair eyelid closure, oral function, and nasal airflow, leading to functional and aesthetic issues. Avoid stigmata!	Dermal substitutes help restore skin texture and elasticity while minimizing contractures or aiding in their removal. Matriderm^®^ or Integra^®^.
Neck	Supports head movement; burns to this region may affect swallowing and breathing.	Scarring may cause contractures that restrict head mobility which could also lead to airway obstruction.	Thin, flexible dermal scaffolds help prevent skin tightness.
Hands & Fingers	Essential in function-grasping, fine motor skills, sensitivity and most of daily activities.	Burns can cause stiffness, vicious scars, tendon exposure or retraction and joint contractures, limiting hand function.	Integra^®^ and collagen-based scaffolds aid in regenerating flexible, durable skin. Matriderm^®^
Axilla (Armpits)	Vital for shoulder movement and stability as well as upper limb function.	Adhesion and contracture formation can limit arm abduction, restricting daily activities.	Mesh grafts and dermal matrices maintain soft, flexible skin.
Feet & Soles	Necessary for walking, balance, and weight distribution.	Scar formation can reduce foot flexibility, stability and may cause pain while walking. Insensate cases are prone to infections or complications.	Fat-enriched skin substitutes improve cushioning and durability.
Joints (Elbows, Knees)	Critical for mobility and range of motion.	Healing skin can become tight and restrictive, leading to contractures.	Silicone-based dermal substitutes help maintain elasticity and prevent stiffness.
Perineum & Genital Area	Essential for urinary, reproductive, and sexual function. Also associate high mortality rate.	High risk of infection, pain, and scarring, leading to functional impairment.	Biological skin substitutes enhance tissue integration and healing.
